# An Assessment of the 10-Year Risk of Developing Type 2 Diabetes Among Saudi Adults Based on the Finnish Diabetes Risk Score

**DOI:** 10.7759/cureus.32034

**Published:** 2022-11-29

**Authors:** Ahmed Elshebiny, Ahmed Alrashed, Zahra Albuwaydi, Sajjad Aljassim, Fatimah Alhammad, Rawan Alhajji

**Affiliations:** 1 Internal Medicine, Diabetes and Endocrinology, Faculty of Medicine, Menoufia University, Shebin El Kom, EGY; 2 Internal Medicine, Diabetes and Endocrinology, King Faisal University, Hofuf, SAU; 3 College of Medicine, King Faisal University, Hofuf, SAU

**Keywords:** body mass index, physical activity, healthy diet, life style, risk factors, saudi arabia, finnish diabetes risk score, types 2 diabetes

## Abstract

Background and objective

Diabetes mellitus (DM) is a chronic, metabolic disease characterized by elevated blood glucose levels that eventually lead to several acute and chronic complications. Type 2 DM (T2DM) is a major healthcare problem globally as well as in the Kingdom of Saudi Arabia (KSA). Predicting and identifying people at high risk for developing T2DM will help implement preventive measures for these individuals. In light of this, the present study was designed to estimate the 10-year risk of developing T2DM among the Saudi general population.

Methodology

A descriptive, cross-sectional survey involving 15,509 Saudi individuals was undertaken. The participants were selected from all 13 provinces of KSA based on stratified random sampling. The Finnish Diabetes Risk Score (FINDRISC), a validated tool for T2DM risk assessment, was employed. Descriptive and chi-square analyses were used.

Results

The mean age of the participants was 28.33 years. Subjects with a moderate, high, and very high risk of developing T2DM within the next 10 years comprised approximately 18% of the sample. The mean FINDRISC was 7.53 [standard deviation (SD): 4.28], which is considered a level associated with a slightly elevated risk of developing T2DM. Of note, 938 participants (6.05%) among the sample population had a high risk of developing T2DM as predicted by FINDRISC. Education, daily physical activity, high blood glucose, and family history of DM were significantly higher in females compared to males (p<0.001). On the other hand, smoking rates and use of antihypertensive medications were substantially higher among males (p<0.001).

Conclusion

Based on our findings, approximately 18% of the Saudi general population has a moderate to high risk of developing T2DM. T2DM risk assessment should be widely and regularly practiced by general practitioners and internists as part of national programs for diabetes prevention.

## Introduction

Diabetes mellitus (DM) is a chronic, metabolic disease characterized by elevated blood glucose levels due to defects in insulin secretion, insulin action, or a combination of both [1,2]. In the past three decades, DM has emerged as one of the top 10 causes of death globally. In 2016, as high as 1.6 million deaths due to diabetes were reported. Worldwide, approximately one in 11 adults have DM. Type 2 DM (T2DM) makes up 90% of all cases of DM [3,4]. It has been estimated that over 50% of DM cases remain undiagnosed. The World Health Organization (WHO) estimated that the number of DM cases is expected to reach 522 million by 2030 [5]. According to the American Diabetes Association (ADA), one in nine people will develop DM by 2030 [6]. A study in 1996 involving 23,493 subjects aged between 20 and 70 years from 34 diverse regions of the Kingdom of Saudi Arabia (KSA) found that the prevalence of type 1 and type 2 DM were 0.217% and 4.993%, respectively. When the population was classified based on gender, the prevalence of type 1 and type 2 diabetes in males was 0.193% and 5.503%, respectively. Whereas in females, the prevalence was 0.237% and 4.556%, respectively [7]. An unhealthy diet and an inactive lifestyle are major risk factors for T2DM. Significantly, several risk factors of T2DM are avoidable, including smoking and alcohol consumption; maintaining body weight by exercise and eating a healthy diet can help too [8]. Complications of DM, including both microvascular and macrovascular complications, could be prevented or delayed [9]. For this purpose, it is important to identify the population at risk by way of risk scoring. Although there are studies that estimate the prevalence of DM among the Saudi Arabian population, there is a dearth of studies in the literature that predict the future risk among the whole KSA population. The prediction of this risk will help to characterize the magnitude of the problem, which will assist in designing and implementing strategies to prevent or treat high-risk cases.

The Finnish Diabetes Risk Score (FINDRISC) is a validated prediction tool for the assessment of the 10-year risk of developing T2DM. The FINDRISC questionnaire, designed by the Finnish Diabetes Association, uses age, body mass index (BMI), physical activity, vegetable and fruit intake, use of antihypertensive medications, history of hyperglycemia, and family history of DM to estimate the 10-year risk of developing T2DM. It does not require any laboratory testing and has been validated in multiple population groups. FINDRISC has been effectively executed as a practical screening tool to evaluate diabetes risk and the development of T2DM [10]. As per this tool, a risk score of 0-6 indicates a low risk (risk of developing T2DM over 10 years). A risk score of 7-11 indicates an elevated risk (4%), and a risk score of 12-14 indicates a moderate risk of diabetes (16.66%). A risk score of 15-20 indicates a high risk of diabetes (33%), and a risk score of >20 indicates a very high risk of diabetes (50%) [10].

The FINDRISC score has been used by different studies, including a study conducted in a health center located in the Harwan district of Srinagar in Jammu and Kashmir, India. It demonstrated a positive link between the FINDRISC score and undiagnosed T2DM among the general population. Moreover, the study revealed that the FINDRISC scoring system could work reasonably well as a screening tool. Moderate to very high risk of T2DM was reported in 22.1% of participants in this study [5]. Another study using FINDRISC was performed in Kuwait, which concluded that 10% of participants were found to have a moderate to very high risk of developing T2DM. Interestingly, 68.7% of the participants in this study were younger than 44 years of age [11]. T2DM represents a significant public health problem. Early recognition of the people at high risk of developing T2DM will pay huge dividends in terms of devising an effective preventive strategy. Moreover, the use of FINDRISC will provide a cost-effective, non-invasive, and reliable risk assessment tool [12]. The current study aims to assess the risk of developing T2DM among the general population in KSA in the next 10 years.

## Materials and methods

Study design and ethical considerations

A cross-sectional study was conducted during the period from April 17, 2021, to May 31, 2021, to assess the risk of developing T2DM among the general Saudi Arabian population in the next 10 years. The study was initiated after obtaining ethical approval from the Institutional Review Board of the King Fahad Hospital, Hofuf (KFHH-IRB), Al-Ahsa, Saudi Arabia (approval number: 19-39-2021). The study followed the tenets of the Declaration of Helsinki on medical research involving human subjects and its amendments. A consent statement with an explanation of the study and its purpose was provided to the participants along with the questionnaire. Additionally, the confidentiality of information was guaranteed.

Sample size

The minimum sample size determined as per the Richard Geiger equation was 385 participants, based on a population size of 20,000,000, a margin of error of 5%, a response distribution of 50%, and a confidence level of 95% [13]. We sought the sample size to be as high as possible to reduce the margin of error. Finally, the study included a sample of 15,509 subjects, which reported the margin of error to be 0.79%.

Inclusion and exclusion criteria

The study included Saudi subjects of both genders who were 18 years or older and did not have an established history of DM. We excluded persons who were under 18 years of age, non-Saudi residents, or established cases of T2DM.

Data collection method

A stratified random sampling method was used to collect the data by distributing an electronic self-administered questionnaire through social media applications. A total of 100 data collectors were involved in the online distribution and collection of data from this large-sized sample from all over the 13 provinces of the KSA. The number of participants from each province ranged from 422 to 1,749. Besides, face-to-face interviews were conducted with some of the participants by a few of the medical students who were involved in collecting data. The research questionnaire was completed by participants by using the FINDRISC, which included data on gender, age, BMI, blood pressure medication use, history of hyperglycemia, physical activity, family history of diabetes, and daily intake of vegetables, fruits, or berries [14-15]. FINDRISC has been validated by many studies and it reportedly has a sensitivity of 81.9% [16]. Additionally, European guidelines have recommended conducting blood tests for T2DM if the score was 15 or more [17].

Statistical analysis

The data analysis was carried out using SPSS Statistics version 26 (IBM, Armonk, NY). Descriptive statistics were used to determine the aspects of sociodemographic data and FINDRISC. Chi-square was used for categorical variables, and a p-value <0.05 was considered statistically significant.

## Results

A total of 15,509 participants were included in the study. The mean age of the participants was 28.33 years [standard deviation (SD): 9.85 years]; 56.9% of them were female while male participants comprised 43.1%. The mean BMI was 25.56 kg/m^2^ while the mean weight was 69.37 kg, and the mean height was 164.39 cm. Among the total participants, 15.2% were smokers; persons who were ever-smokers accounted for 7%, and those who never smoked represented 77.6%, as shown in Table 1. Table 2 shows the distribution of the study subjects by geographical area. The number of participants from each province ranged from 422 from the Province of the Northern Borders to 1,749 from Riyadh.

**Table 1:** Sociodemographic data of the study population (n=15,509) BMI: body mass index; IQR: interquartile range; SD: standard deviation

Variable	N	%
Age, years		
<45	14,050	90.6
45–54	1,149	7.4
55–64	266	1.7
>64	44	0.3
Range	18.0–90.0	
Mean ± SD	28.33 ± 9.85	
Median (IQR)	24.0 (21.0–33.0)	
Gender		
Male	6,681	43.1
Female	8,828	56.9
Marital status		
Married	5,625	36.3
Single	9,506	61.3
Divorced	286	1.8
Widowed	92	0.6
Education level		
None/primary	104	0.7
Intermediate	1,736	11.2
Secondary	4,168	26.9
University	9,501	61.3
Smoking status		
Smoker	2,363	15.2
Ever smoker	1,111	7.2
Never smoker	12,035	77.6
BMI, kg/m^2^		
<25	8,153	52.6
25–30	4,192	27
>30	3,164	20.4
Range	12.63–55.88	
Mean ± SD	25.56 ± 5.81	
Median (IQR)	24.65 (21.39–28.76)	

**Table 2:** Distribution of the study participants by geographical area (across the 13 Saudi provinces)

Province	N	%
Al Bahah	956	6.2
Al Jouf	1,161	7.5
Northern Borders	422	2.7
Riyadh	1,749	11.3
Eastern Province	1,723	11.1
Al-Qassim	1,469	9.5
Medina	1,113	7.2
Tabuk	1,185	7.6
Jazan	1,206	7.8
Ha'il	927	6
Asir	1,151	7.4
Makkah	1,555	10
Najran	892	5.8

As shown in Table 3, 87.8% of men had waist circumferences of 102 cm or less, and 89% of women had waist circumferences of less than 88 cm. Overall, a total of 8,656 participants (55.8%) reported daily physical activity of 30 minutes or more. Surprisingly, nearly 56 individuals reported not consuming vegetables, fruit, or berries daily. The majority of subjects denied using antihypertensives (96.2%), while 593 individuals admitted to using them (3.8%). The study showed that 1,298 subjects (8.4%) had a history of high blood glucose. Almost three-quarters (n=11,355; 73.1%) of the respondents had a family history of diabetes.

**Table 3:** Responses to FINDRISC questionnaires by the study participants FINDRISC: Finnish Diabetes Risk Score

	N	%
Waist circumference, cm		
Men		
>94	3,772	56.50
94–102	2,091	31.30
<102	818	12.20
Women		
>80	5,102	57.80
80–88	2,750	31.20
<88	976	11.10
Daily physical activity, 30 minutes or more		
No	6,853	44.2
Yes	8,656	55.8
Daily vegetable, fruit, or berry consumption		
No	8,637	55.7
Yes	6,872	44.3
Antihypertensive use		
No	14,916	96.2
Yes	593	3.8
History of high blood glucose		
No	14,211	91.6
Yes	1,298	8.4
Family history of diabetes		
No	4,174	26.9
First relative	7,034	45.4
Second relative	4,301	27.7

Table 4 demonstrates the differences between male and female participants with regard to sociodemographic characteristics including educational level and BMI. Males were significantly older than females (p<0.001), while education level was higher among females (p<0.001). Smoking prevalence was significantly higher in men (p<0.001), and BMI was significantly higher among males than females (p<0.001).

**Table 4:** Comparison between male and female participants regarding sociodemographic characteristics *Statistically significant BMI: body mass index; IQR: interquartile range; SD: standard deviation

	Male (n=6,681)		Female (n=8,828)		Test (p-value)
	N	%	N	%	
Age, years					
<45	5,977	89.5	8,073	91.4	X^2^=91.290 (<0.001*)
45–54	490	7.3	659	7.5	
55–64	190	2.8	76	0.9	
>64	24	0.4	20	0.2	
Range	18.0–90.0		18.0–89.0		Student's t-test=5.643 (<0.001*)
Mean ± SD	28.85 ± 10.21		27.94 ± 9.56		
Median (IQR)	25.0 (22.0–33.0)		24.0 (21.0–34.0)		
Marital status					
Married	2,254	33.7	3,371	38.2	X^2^=150.06 (<0.001*)
Single	4,360	65.3	5,146	58.3	
Divorced	52	0.8	234	2.7	
Widowed	15	0.2	77	0.9	
Education level					
None/primary	35	0.5	69	0.8	X^2^=127.31 (<0.001*)
Intermediate	879	13.2	857	9.7	
Secondary	1,995	29.9	2,173	24.6	
University	3,772	56.5	5,729	64.9	
Smoking status					
Smoker	2,154	32.2	209	2.4	X^2^=3838.1 (<0.001*)
Ever smoker	932	14	179	2	
Never smoker	3,595	53.8	8,440	95.6	
BMI, kg/m^2^					
<25	3,061	45.8	5,092	57.7	X^2^=215.2 (<0.001*)
25–30	2,078	31.1	2,114	23.9	
>30	1,542	23.1	1,622	18.4	

Table 5 shows the FINDRISC questionnaire responses from the study population by gender. Physical activity, history of high glucose, and family history of DM were significantly higher in women than in men (p<0.001). On the other hand, smoking and antihypertensive use were significantly higher among males (p<0.001). However, no significant differences were found with regard to daily vegetable, fruit, or berry consumption.

**Table 5:** Association between gender and FINDRISC questionnaire responses among the study participants *Statistically significant FINDRISC: Finnish Diabetes Risk Score

	Male (n=6,681)		Female (n=8,828)		X^2^ (p-value)
	N	%	N	%	
Daily physical activity, 30 minutes or more					
No	3,107	46.5	3,746	42.4	X^2^=25.567 (<0.001*)
Yes	3,574	53.5	5,082	57.6	
Daily vegetable, fruit, or berry consumption					
No	3,758	56.2	4,879	55.3	X^2^=1.485 (0.223)
Yes	2,923	43.8	3,949	44.7	
Antihypertensive use					
No	6,388	95.6	8,528	96.6	X^2^=10.080 (0.001*)
Yes	293	4.4	300	3.4	
History of high blood glucose					
No	6,258	93.7	7,953	90.1	X^2^=63.565 (<0.001*)
Yes	423	6.3	875	9.9	
Family history of diabetes					
No	2,046	30.6	2,128	24.1	X^2^=133.74 (<0.001*)
Second relative	1,575	23.6	2,726	30.9	
First relative	3,060	45.8	3,974	45	

**Figure 1:**
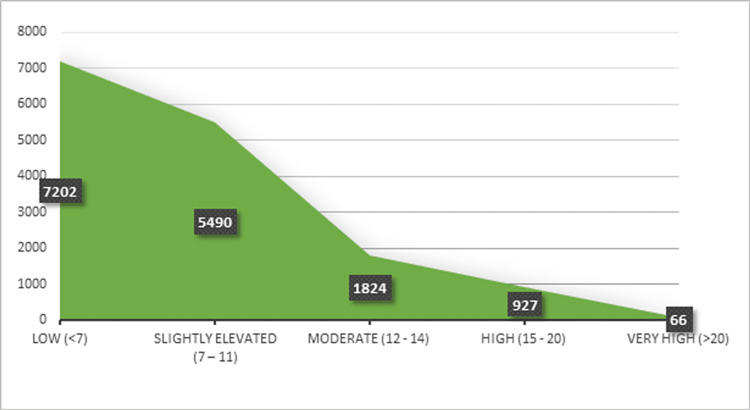
Distribution of FINDRISC risk levels among the study population FINDRISC: Finnish Diabetes Risk Score

Figure 1 shows the distribution of FINDRISC risk levels among the study population and the probability of developing T2DM by each risk level. Subjects with a moderate, high, and very high risk of developing T2DM within the next 10 years comprised approximately 18% of the sample population. The mean FINDRISC was 7.53 (SD: 4.28), which is considered a level associated with a slightly elevated risk of developing T2DM. Of note, 938 participants (6.05%) among the sample population had a high risk of developing T2DM as predicted by FINDRISC.

## Discussion

To the best of our knowledge, this is the first study to estimate the 10-year risk for developing T2DM based on FINDRISC among the general population of Saudi Arabia covering all provinces of the Kingdom. Of note, the sample was randomized and included subjects from all 13 provinces of KSA in a stratified manner. The sample of this survey was quite high, reaching more than 15.5K responses, to maximize the precision of the results and give a broader idea of the 10-year risk of DM spanning the whole Kingdom.

The study results showed that approximately one-fifth (18%) of the population showed a moderate, high, or very high risk of developing T2DM within the next 10 years. The mean FINDRISC was found to be 7.53 (SD: 4.28), which reflects a mildly elevated level of potential risk among the study population. In a study conducted in Harwan involving 1,530 participants based on FINDRISC, the mean score was reported as 11.45 (SD: 4.8) [5]. Another study in Hungary using FINDRISC found the mean to be 10.45 ± 5.09 [18]. The median risk score was 9.0 in a study from Kuwait [11]. In 2010, a cross-sectional study conducted in the Al-Qassim region of Saudi Arabia involving 2,007 subjects did not report FINDRISC mean scores [19].

Moreover, the mean age was 37.7 years in the study performed in Kuwait, while it was 50.4 years in the Harwan study, and 60 years in the study from Hungary; meanwhile, our study reported a mean age of 28.3 years. Age may have been a significant factor in terms of the increase in FINDRISC in the mentioned studies [5,11,18]. In the current study, nearly 1,000 participants (6.05%) were considered at high risk of developing T2DM as per FINDRISC. In the previously mentioned study conducted in Al-Qassim, KSA, 8.68% (174) of the sample was determined to develop T2DM [19].

In the current study, regarding the reported T2DM risk factors, the findings showed that about 9/10 of the participants’ waist circumferences were less than 102 cm among men and less than 88 cm among women. More than half of the participants reported daily physical activity, and more than half did not consume vegetables, fruits, or berries daily. Compared to this study, a study done in Kuwait showed that waist circumferences of 80% of men were less than 102 cm and that of 59% of women were less than 88 cm. Regarding physical activity and consumption of fruits and vegetables, the numbers in the Kuwait study were 60.8% and 47.3 respectively, which paints a better picture as compared to our study [11]. In the Harwan study, researchers found that 87.4% of subjects had waist circumferences of less than 102 cm and less than 88 cm for men and women, which more or less aligns with our results. In addition, 96.4% of the participants in the Harwan study reported daily physical activity [5]. This is in contrast with the findings among Italian and Spanish people in a cross-sectional study that involved 32,722 individuals, which reported that only 12.2% of people were physically active in Italy and only 16.5% of the participants were active in Spain. Of note, 75.7% of both Spanish and Italian participants reported daily consumption of vegetables, fruits, or berries [12].

Interestingly, most of the current study respondents were not using antihypertensive medications (96.2%). A history of high blood glucose was not common among the study participants. Moreover, almost three-quarters (n=11,355; 73.1%) of respondents had a family history of diabetes. This reflects a high prevalence of established DM cases in the Saudi population. In contrast, the study among Italian and Spanish people found that while 31.6% used antihypertensive drugs, only 1/10 participants reported previous high blood glucose levels [12]. Only 42.8% had a family history of DM. Another study by Enikuomehin et al., conducted among doctors in the Ondo state in Nigeria, revealed that only 25.5% of the participants had a family history of DM [20].

To summarize, the findings of the current study, which aimed to assess the 10-year risk of developing T2DM among a huge sample of relatively young Saudi citizens, showed alarming results. Hopefully, the implementation of appropriate diabetes preventive measures would help improve the situation.

This study has a few limitations. Primarily, this study was based on self-reported data such as diagnosis of diabetes, medication use, and physical activity, which may have allowed certain biases to creep in. However, we believe that this shortcoming is overcome by the large size of the sample.

## Conclusions

Based on our findings, approximately 18% of the Saudi population has a moderate to high risk of developing T2DM. Of them, 6% of the total study population has a high risk of developing T2DM. These results are alarming and healthcare policymakers should plan and implement new policies for targeted preventive care of T2DM. Risk assessment for T2DM should be widely and regularly practiced by general practitioners and internists as part of national programs for diabetes prevention.
